# Effect of Calcium Hydroxide Versus Double Antibiotic Paste on Endodontic Treatment Outcomes in Teeth With Large Periapical Lesions: A Triple-Blind Randomized Clinical Trial

**DOI:** 10.1155/ijod/7071459

**Published:** 2024-12-23

**Authors:** Afsaneh Rahmati, Farshad Seyedein, Omid Dianat, Sara Saedi, Golriz Rostami, Alireza Akbarzadeh Baghban, Shima Sabertahan, Majid Kazem

**Affiliations:** ^1^Endodontic Department, Hamedan University of Medical Sciences Dental School, Hamedan, Iran; ^2^Endodontist, Private Office, Tustin, California, USA; ^3^Endodontic Department, University of Maryland Dental School, Baltimore, Maryland, USA; ^4^Endodontist, Private Office, Tehran, Iran; ^5^Department of Biostatistics, Shahid Beheshti University of Medical Sciences, Tehran, Iran; ^6^Department of Endodontics, Shahid Beheshti University of Medical Sciences Dental School, Tehran, Iran

**Keywords:** antibiotics, calcium hydroxide, periapical periodontitis, root canal medicaments

## Abstract

**Objective:** The successful management of necrotic pulps and apical periodontitis poses a tough challenge in endodontic therapy, as it involves addressing compromised tooth vitality and microbial invasion of root canal systems. Failure to effectively treat these conditions can lead to persistent infection and severe patient discomfort. The efficacy of double antibiotic paste (DAP), a mixture of ciprofloxacin and metronidazole, was evaluated and compared to calcium hydroxide (CH) by assessing radiographic and clinical outcomes of nonsurgical endodontic treatment in cases with necrotic pulps and the presence of apical periodontitis.

**Methods:** Thirty maxillary anterior teeth with necrotic pulps and apical periodontitis (periapical index [PAI] = 5) were divided randomly into two experimental groups. The first group received CH dressing, while the other group filled with DAP as intracanal medicament. After 3 weeks, medicaments were removed, and teeth were obturated in both groups. Teeth were assessed clinically and radiographically at 6-month and 12-month follow-ups. A chi-square test was performed to compare the outcome between the groups.

**Results:** None of the teeth showed complete healing in a 6-month follow-up. At the 12-month follow-up, six samples in the DAP group showed complete healing of the periapical (PA) lesion, while none of the samples in the CH group were completely healed. The PA healing outcome was as follows: in the DAP group, 40% of cases were healed, 60% healing, and 0% diseased, while in the CH group, 0% healed, 93.3% healing, and 6.7% diseased. The results of the two experimental groups in 12 months differed significantly (*p*  < 0.05).

**Conclusion:** DAP group has shown significantly better outcomes compared to CH as intracanal medicament in the treatment of teeth with PA lesions. This finding suggests that DAP may offer a more effective therapeutic approach in managing necrotic pulps and apical periodontitis, potentially improving patient outcomes and treatment success rates.

**Trial Registration:** Clinical Trial Registry identifier: IRCT2015060822615N1

## 1. Introduction

Bacterial elimination from the root canal system is essential for periapical (PA) healing. However, complete disinfection has remained a challenge in teeth with necrotic pulps and apical periodontitis due to the polymicrobial nature of infection and the complex anatomy of the root canal [1, 2]. Despite the substantial reduction in the communities of bacteria after chemomechanical preparation, cultivable bacteria were detected in 40%–60% of canals prepared and irrigated with sodium hypochlorite [3, 4]. The application of intracanal medications has shown additional disinfection of the root canal system [5]. Given that reconstructing occlusion in edentulous mouths is very difficult and complex, every effort should be made to preserve the patients' teeth [6]. Devitalized teeth, with altered moisture content and dentin composition, are more prone to fractures and lack natural defense compared to vital teeth. Weakened bonding between restorations and tooth structure exacerbates risks of recurrent infection and fractures. Recognizing chemical and biomechanical distinctions is crucial for treatment planning, focusing on reducing bacterial loads and meticulous restoration design to enhance long-term outcomes, success, and functionality [7].

The limited antimicrobial effectiveness of calcium hydroxide (CH), the most common interappointment medicament—against microorganisms, most importantly, those involved in endodontic failures—has become a matter of concern [8]. Triple antibiotic paste (TAP), the mixture of ciprofloxacin, metronidazole, and minocycline, has shown efficiency in disinfecting the root canal system in specific concentrations [9]. TAP is effective against gram-negative, gram-positive, and anaerobic bacteria in odontogenic infections [10]. A recent systematic review of in vitro studies showed that antibiotic pastes were either superior or equal to CH [11]. Its application as an intracanal medication for the healing of PA lesions has been reported and advocated by several clinical studies [12–15]. One major drawback of TAP is tooth discoloration caused by the minocycline component [16].

To overcome this issue, minocycline has been eliminated from the formulation to produce a double antibiotic paste (DAP). Its antimicrobial efficacy is comparable to TAP [7, 15] and has been successfully used in clinical cases to treat large PA lesions [16, 17]. Furthermore, CH and DAP do not hurt root fracture resistance when used as intracanal medicaments for less than 1 month [18]. To date, based on our knowledge, no clinical trials have compared the efficiency of DAP and conventional CH therapy on the 12-month outcome of nonsurgical root canal treatment of teeth with apical periodontitis. Therefore, this triple-blinded randomized clinical trial was designed to clinically and radiographically compare the efficacy of CH and DAP as intracanal medicaments on the healing outcome of anterior maxillary teeth with apical periodontitis. The null hypothesis was the equivalency of the outcome variable distribution between the two experimental groups or the independency of the outcome variable and the experimental group variable.

## 2. Materials and Methods

### 2.1. Study Setting

The study design was a parallel randomized controlled trial. The protocol of this study was approved by the Ethics Committee of the Shahid Beheshti University of Medical Sciences (Code: *ir.sbmu.rids.rec.1394.181*). This study was conducted in accordance with the CONSORT 2010 Statement [19]. Patients were informed about the nature of this study, associated risks, and the required follow-up periods, and written informed consent forms were signed before initial treatment.

### 2.2. Participants

In total, 30 patients referred to the Endodontic Department, Shahid Beheshti University of Medical Sciences School of Dentistry, in the duration between March 2018 and March 2020, who met the following criteria were included in this study:

• Age between 18 and 30 years
• Having one nonvital anterior maxillary tooth with the presence of apical periodontitis
• Lesion size >5 mm
• PA index (PAI) = 5 [17]

The exclusion criteria were defined as follows:

• Patients with presence of systemic diseases
• Allergy to the antibiotics (metronidazole or ciprofloxacin)
• Patients not able to take oral NSAIDs
• Pregnancy or nursing
• Vertical root fractures
• Calcified canals
• Internal/external root resorptions
• Roots with open apices
• Nonrestorable teeth
• Patients with masticatory parafunctions

Patients were randomly assigned into two groups (*n* = 15/group) in a 1:1 ratio in blocks of 2, based on the intracanal medication used. Randomization was performed using online software at www.randomization.com. The assistant supervisor generated the random sequence and assigned the participants to same covered capsules of intracanal medicament, either CH or DAP. This study was a triple-blind study, and the sequence remained blinded to all team members throughout the entire treatment and follow-up course.

### 2.3. Preoperative Examinations

Each tooth was clinically examined for sensitivity to palpation, percussion, presence of sinus tract, or intra-/extraoral swelling and abscess. Preoperative radiographs were taken using the RINN XCP system (Dentsply, North Carolina, USA) by paralleling technique with the digital radiovisiography (RVG) (Suni Ray, Suni Imaging Microsystem Inc., California, USA).

### 2.4. Intervention

All patients accepted the two-visit root canal treatment. All clinical procedures were performed by a single postgraduate student.

### 2.5. First Visit

After local anesthesia and proper rubber dam isolation, access cavities were prepared, and the working length was established using a Raypex 5 Apex locator (VDW, Munich, Germany) 1 mm short of apical constriction and then further confirmed using radiographs. The root canals were prepared using rotary BioRace files (FKG Dentarie SA, Chax-de-Fonds, Switzerland) to file 35, 4% along with 10 cc irrigation with 2.5% sodium hypochlorite (Cerkamed, Stalowa Wola, Poland) irrigation by special endodontic needle gauge #30 and 1-min ultrasonic passive irrigation.

In cases with the presence of intraoral swelling, intracanal drainage was achieved by passing a small k-file #10 or 15 (Mani, Inc., Tokyo, Japan) 1 mm beyond the apex. Subsequently, to remove the smear layer, 1 mL of EDTA 17% (Cerkamed, Stalowa Wola, Poland) irrigation followed by 5 mL NaOCl 2.5% was applied. The canals were dried and filled with intracanal medication plugged into the canal by using Lentulo spiral according to the randomly selected group (DAP group—a combination of 500 mg ciprofloxacin, Exir, Borujerd, Iran) and 500 mg metronidazole (Chemidarou, Tehran, Iran) ground and then mixed with 2 cc saline to obtain creamy consistency 500 mg/cc and CH (Cerkamed, Stalowa Wola, Poland) group, creamy consistency 500 mg/cc).

A layer of 4-mm interim restoration material (IRM) (3 M ESPE Dental, Minnesota, USA) was used to obtain a tight coronal seal. The analgesic regimen of ibuprofen 400 mg every 6 h was prescribed. No systemic antibiotics were prescribed.

### 2.6. Second Visit

After 3 weeks of medication, the patients were re-evaluated for signs and symptoms. The canals were re-entered under rubber dam isolation, and the intracanal medications were removed using 5 mL of NaOCl 2.5% and circumferential filing with a Hedstrom file. The canals were dried and obturated with gutta-percha and AH 26 sealer (Dentsply, De Trey, Konstanz, Germany) using a lateral condensation technique. The teeth were restored permanently with composite fillings within 2 weeks after the obturation session.

### 2.7. Follow-Up Examination

The patients were recalled for evaluation 6 and 12 months after treatment. Clinical and radiographic examinations were performed in follow-up sessions. Radiographs were taken using a parallel technique, and the postoperative PAI index was independently reported by two blinded examiners (both board-certified endodontists). In cases of disagreements on the evaluation of the PA status, the two examiners discussed the conflict to report a unique answer. Based on clinical and radiographic presentations, the outcome was defined as healed/healing/diseased [18].

The “healed” category defined both normal clinical and radiographic presentations.

The “healing” category defined normal clinical presentation with a reduced PA radiolucency size.

The “diseased” category stands for unchanged or emerged PA radiolucency with/without clinical signs/symptoms.  Table 1 presents the PAI criteria defined by Ørstavik D [17].

Comparing the preoperative PAI (PAI = 5 for all samples) and postoperative PAI, the samples were categorized as follows based on radiographic evaluation [15]:

• Healed: PAI 5 changed to PAI 1.2.
• Healing: PAI 5 changed to PAI 3.4.
• Diseased: PAI 5 remained unchanged postoperatively.

### 2.8. Sample Size and Statistical Analysis

The sample size was calculated to be 30 based on type 1 error *α* = 0.05, type 2 error *β* = 0.2 (power = 80%), and effect size = 0.56 according to the chi-square test. Adequate sample size was *n* = 30 (15 in each group).

The results were analyzed using SPSS software version 21(SPSS Inc., Chicago, IL) by a blinded statistician. The chi-square test was used to compare the results between the two groups. The level of significance was set at *p*  < 0.05. The estimated effect size and 95% confidence interval are presented in Table 2.

## 3. Results

### 3.1. First Appointment

A summary of the preoperative conditions of samples in both groups is presented in Table 3. The distribution of preoperative factors between the two groups showed no significant differences using the chi-square test. In seven cases with intraoral swelling, drainage through the root canal was achieved, and there was no further need for incision and drainage and systemic antibiotics.

### 3.2. Second Appointment

Clinical assessments in this session showed that the teeth in both groups were symptom-free, with no pain in percussion and palpation. No intra-/extraoral swellings were observed. No teeth had sinus tract except one tooth in the CH group, which had a nondraining sinus tract with a history of previous swelling on day 0.

### 3.3. Six-Month Follow-Up

The same tooth in the CH group, with an inactive sinus tract on the second visit, still presented the sinus tract, with no changes in the size of the PA lesion. This tooth was considered “diseased.” The patient was informed of the root canal failure, and surgical root canal treatment was further performed. All other samples in CH and DAP groups were categorized as “healing.” Complete healing of the PA lesion was not observed in any teeth after 6 months.  Table 4 shows the healing status of teeth in both experimental groups after 6 months. According to the chi-square test, there is no significant difference in healing between the two groups (*p*=1.000).

### 3.4. Twelve-Month Follow-Up

At the 12-month follow-up, six of the teeth in the DAP group were completely healed, while in the CH group, none of the teeth were completely healed. The summary of the healing status of teeth in each group after 12 months is presented in Table 5.

**Figure 1:**
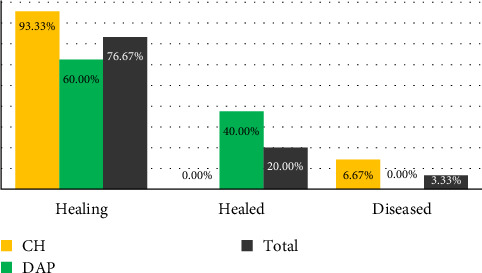
Healing outcome of endodontic treatment in both groups. CH, calcium hydroxide; DAP, double antibiotic paste.

The outcome of nonsurgical root canal treatment using intracanal medications is presented in Figure 1. The results of endodontic treatment were compared in the two treatment groups using the chi-square test. Based on the results of this statistical test, a significant difference in healing was reported between the two groups after 12 months of follow-up (*p*=0.017).

**Figure 2:**
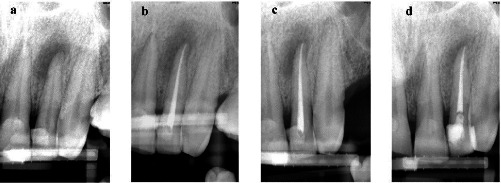
“Healing” lesion in the calcium hydroxide (CH) group: (a) preoperative radiograph of tooth #10 showing a large apical lesion with periapical index (PAI) 5; (b) immediate postoperative radiograph; (c) 6-month follow-up with evidence of a reduction in the size of the lesion; and (d) 12-month follow-up with significant healing of the lesion, slight apical radiolucency is still evident.

**Figure 3:**
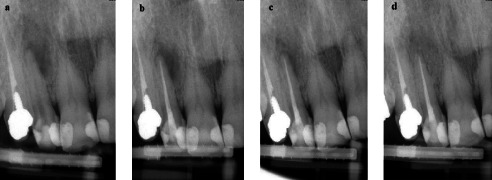
“Healed” lesion in the double antibiotic paste (DAP) group: (a) preoperative radiograph of tooth #7 showing a large apical lesion with periapical index (PAI) 5; (b) immediate postoperative radiograph; (c) 6-month follow-up showing significant reduction in the size of the lesion; (d) 12-month follow-up with intact lamina dura formation and no evident radiolucency, presenting the normal status of the periapical region.

Figures 2 and 3 present the healing process of cases treated with CH and DAP with different outcomes after 12 months.

## 4. Discussion

This randomized clinical trial with a 12-month follow-up was designed to evaluate the outcome of two-visit nonsurgical root canal therapy using DAP or CH as the intracanal medicaments in teeth with PA lesions. Single-rooted anterior maxillary teeth were treated with a similar chemomechanical preparation to eliminate the confounding factors. The outcome was evaluated with the criteria for determination of PA status defined by Friedman and Mor [18].

TAP, a combination of ciprofloxacin, metronidazole, and minocycline antibiotics, was introduced by Hoshino et al. [20] as an antibiotic paste with sufficient potential for eradication of bacteria from dentin of infected root canals [20, 21]. It has been widely used as a disinfecting agent in immature necrotic teeth with apical periodontitis undergoing revascularization procedures [22]. The American Association of Endodontics (AAE) Regenerative Committee recommended the use of TAP, DAP, or CH as intracanal medicament after chemical debridement [9].

Besides the application of antibiotic pastes in regenerative endodontics, the use of these antibiotic combinations as intracanal medication in mature teeth has been reported in cases of large PA lesions [10] and to avoid endodontic flare-ups in diabetic patients [23]. In a randomized clinical trial performed on teeth with necrotic pulp and apical periodontitis, Arruda and colleagues reported a 97% reduction in total bacteria after using triple antibiotic solution as intracanal medication following chemomechanical preparation. However, this bacterial reduction was only 39% for the CH-treated teeth. This finding can explain our results in the present study to have better outcome in antibiotic group [13]. Taneja and colleagues reported a series of cases with failure and persistence of infection with CH intracanal medication [23]. After 3 weeks, the intracanal medication was replaced with TAP in these cases, and the relief of symptoms and complete healing were observed during follow-ups. In our study, we had similar results of healing in antibiotic group. To conclude, it was suggested that when common intracanal medications fail to eradicate infections, the clinical use of intracanal antibiotic pastes should be considered as an alternative [12, 24]. In another clinical trial, Johns et al. [15] clinically and radiographically compared the outcome of treatment in teeth with large PA lesions using TAP, CH, and photodynamic therapy (PDT) for disinfection. They reported 15% failure in the CH group and 5% failure in the TAP group at the 18-month follow-up. This investigation demonstrated a superior efficacy of antibiotics compared to CH, a correlation consistent with the findings of the present study. To summarize the highlights of the present study, at the 6-month follow-up, none of the samples in both groups showed healed PA status. At the 12-month follow-up, six samples in the DAP group were categorized as “healed,” whereas no samples in the CH group were in the “healed” category. Moreover, the PA status of only one sample, in the CH group, was stated as “diseased” at the 12-month follow-up. Statistical analysis showed significant differences between the groups in 12-month follow-up periods; based on the results of this study, it can be concluded that DAPs have better long-term outcomes in the healing process of PA lesions. The higher level of healing in the antibiotics group in this study is to the previous studies mentioned.

DAP was alternatively used in this study for the anterior teeth of patients, considering that TAP has the potential for tooth discoloration. DAP has shown comparable antimicrobial effectiveness to TAP in in vitro studies [11, 22, 25]. TAP and DAP significantly reduced *E. faecalis* CFU/mL when compared to CH [12, 26]. In another study, Latham et al. [9] reported the most significant overall reduction of bacterial count in extracted teeth with TAP and DAP at the concentration of 10 mg/mL in comparison to CH. These findings are also in agreement with the results of our current study.

In the current study, digital PA radiography was used for radiographic evaluation of the PA status. This is a two-dimensional imaging modality used for many years in endodontics to evaluate the outcome of treatment. Based on a recent systematic review, digital PA radiography has an accuracy value of 0.72 which is considered a good diagnostic accuracy [27]. Cone-beam computed tomography (CBCT) is the three-dimensional imaging modality, which has recently been used for outcome assessment of endodontic treatments in cases with apical periodontitis [28]. It has an excellent diagnostic accuracy of 0.96 [27]. It was reported by Torabinejad et al. [29] that 20% of teeth with successful root canal treatments based on conventional radiographs had CBCT lesions measuring greater than 1 mm. The clinicians were cautioned for overtreatment of these lesions, and longer follow-up periods were recommended. Therefore, as a limitation of this study, it could be mentioned that evaluating the process of PA healing would have been more accurate and relevant using the CBCT technology and should be considered in further clinical trials. Additionally, the limited sample size of the study should also be mentioned, which accentuates the need for further studies to confirm the effectiveness of DAP as an intracanal medication in the outcome of root canal endodontic treatment.

### 4.1. Limitations of the Study

• Short follow-up period because of the COVID-19 pandemic: The follow-up duration of 12 months might not fully capture long-term treatment outcomes.
• Radiographic evaluation: Reliance on digital PA radiography may have limitations compared to more advanced imaging techniques like CBCT.
• Single-center study: Conducted at a single center, which may limit the generalizability of the findings to broader clinical settings.

Addressing these limitations in future studies could enhance the reliability and applicability of the research findings.

## 5. Conclusion

Based on the results of the present clinical trial, it can be concluded that DAP as an intracanal medication has a trend of better healing outcomes compared to conventional CH therapy. Further studies with greater sample sizes and using CBCT for outcome assessment are recommended. Comparison of outcomes for longer periods might also affect the conclusions and must be further considered.

## Figures and Tables

**Table 1:** PAI scoring criteria.

PAI score	Criteria
1	Normal periapical structures
2	Small changes in bone structure
3	Changes in bone structure with some mineral loss
4	Periodontitis with a well-defined radiolucent area
5	Severe periodontitis with exacerbating features

Abbreviation: PAI, periapical index.

**Table 2:** Estimated effect size and 95% confidence interval for P_2(DAP)_–P_1(CH)_.

PA radiographic evaluation	Effect size (P2–P1)	Lower bound	Upper bound
Healed	0.400	0.152	0.648
Healing	−0.333	−0.611	0.000
Diseased	—	—	—

**Table 3:** Preoperative clinical findings of cases in each treatment group.

Clinical findings	CH	DAP
Pain on percussion	3/15	4/15
Pain on palpation	3/15	5/15
Sinus tract	2/15	1/15
Intraoral swelling	3/15	4/15
Extraoral swelling	0/15	0/15

Abbreviations: CH, calcium hydroxide; DAP, double antibiotic paste.

**Table 4:** The 6-month outcome of endodontic treatment.

PA radiographic evaluation	CH	DAP
Healed	0/15	0/15
Healing	14/15	15/15
Diseased	1/15	0/15

*Note:* Healing status is summarized for each treatment group. There is no significant difference in healing between the two groups (*p*=1.000).

Abbreviations: CH, calcium hydroxide; DAP, double antibiotic paste.

**Table 5:** The 12-month outcome of endodontic treatment.

PA radiographic evaluation	CH	DAP
Healed	0/15	6/15
Healing	14/15	9/15
Diseased	1/15	0/15

*Note:* A significant difference in healing was reported between the two groups (*p*=0.017).

Abbreviations: CH, calcium hydroxide; DAP, double antibiotic paste.

## Data Availability

The data that support the findings of this study are available on request from the corresponding author.
